# Iterative Exponential Growth of Oxygen-Linked Aromatic Polymers Driven by Nucleophilic Aromatic Substitution Reactions

**DOI:** 10.3389/fchem.2021.620017

**Published:** 2021-04-28

**Authors:** Tyler J. Jaynes, Mona Sharafi, Joseph P. Campbell, Jessica Bocanegra, Kyle T. McKay, Kassondra Little, Reilly Osadchey Brown, Danielle L. Gray, Toby J. Woods, Jianing Li, Severin T. Schneebeli

**Affiliations:** ^1^ Department of Chemistry, University of Vermont, Burlington, VT, United States; ^2^ George L. Clark X-Ray Facility and 3M Materials Laboratory, University of Illinois at Urbana-Champaign, Urbana, IL, United States

**Keywords:** iterative convergent/divergent polymer synthesis, SNAr reactions, nonclassical hydrogen bonding, nuclear magnetic resonance spectroscopy, polymer folding, iterative exponential polymer growth, transition metal-free coupling

## Abstract

This work presents the first transition metal-free synthesis of oxygen-linked aromatic polymers by integrating iterative exponential polymer growth (IEG) with nucleophilic aromatic substitution (S_N_Ar) reactions. Our approach applies methyl sulfones as the leaving groups, which eliminate the need for a transition metal catalyst, while also providing flexibility in functionality and configuration of the building blocks used. As indicated by 1) ^1^H-^1^H NOESY NMR spectroscopy, 2) single-crystal X-ray crystallography, and 3) density functional theory (DFT) calculations, the unimolecular polymers obtained are folded by nonclassical hydrogen bonds formed between the oxygens of the electron-rich aromatic rings and the positively polarized C–H bonds of the electron-poor pyrimidine functions. Our results not only introduce a transition metal-free synthetic methodology to access precision polymers but also demonstrate how interactions between relatively small, neutral aromatic units in the polymers can be utilized as new supramolecular interaction pairs to control the folding of precision macromolecules.

## Introduction

**FIGURE 1:**
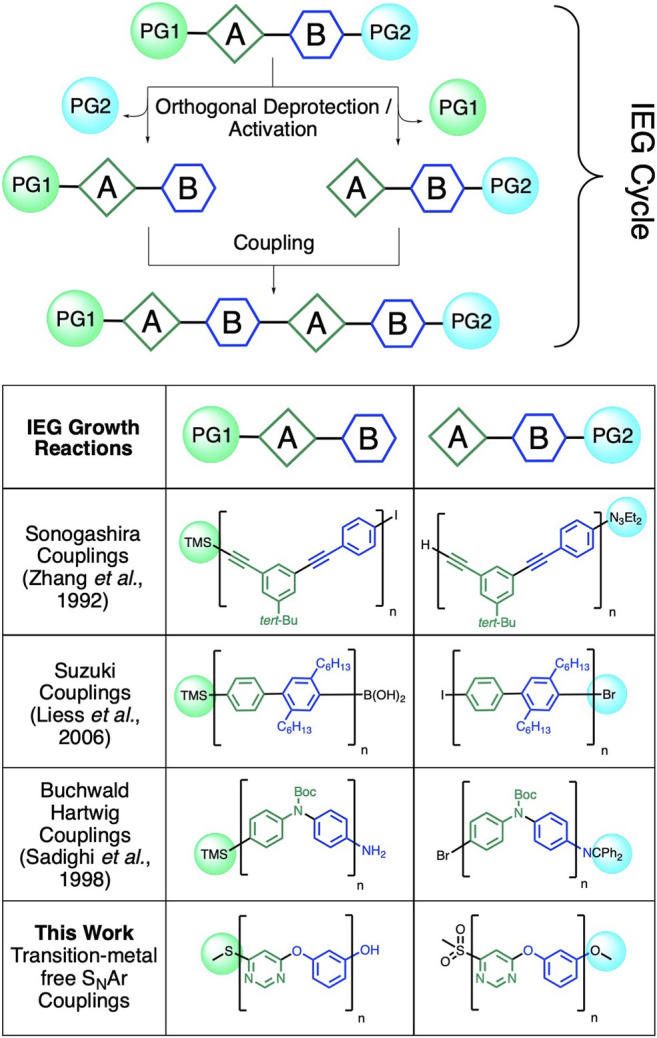
Comparison of previously reported transition metal (TM)-catalyzed IEG methods to our TM-free strategy for the construction of well-defined polyaromatic precision macromolecules. Our approach eliminates the need for transition metal catalysts by introducing methyl sulfones as efficient leaving groups for S_N_Ar-based IEG processes.

The backbones of conjugated and heteroatom-linked aromatic polymers tend to possess fewer conformational degrees of freedom than polymers with more flexible aliphatic or partially aliphatic backbones. This reduced amount of conformational freedom can help enhance the folding of aromatic polymers, to advance a variety of useful properties such as selective supramolecular recognition (Goodman et al., 2007; Liu et al., 2015; Otte, 2016; Schneebeli et al., 2016; Adhikari et al., 2017; Xie et al., 2020), selective catalysis (Rajappan et al., 2020; Sharafi et al., 2020), and self-assembly (Cole et al., 2017; Greene et al., 2017; Bonduelle, 2018; Ong and Swager, 2018; Delawder et al., 2019; Zhao et al., 2019). However, while the precise chemical structures, lengths, and sequences of such macromolecules (Dobscha et al., 2019; Gerthoffer et al., 2020; Zhao et al., 2020) dictate their folding, and with it their functionalities and physical properties (Chen et al., 2015; Hanlon et al., 2017), it remains challenging to synthesize polyaromatic structures with precise lengths and/or sequences as unimolecular entities. One of the most efficient ways to precisely control the length and sequence of synthetic polymers is by iteratively coupling (Jones et al., 1997) polymer strands together in a convergent/divergent fashion (Hawker et al., 1997; Read et al., 2000; Grayson and Frechet, 2001; Li et al., 2005; Liess et al., 2006; Binauld et al., 2011). This methodology (Figure 1) is generally referred to as iterative exponential growth (IEG) (Barnes et al., 2015; Leibfarth et al., 2015).

IEG requires a dormant monomer that includes two distinct functionalities, which are orthogonally deprotected/activated for coupling, to afford two reactive species that can be coupled together selectively. These two reactive species are then combined in a chemo-selective manner, resulting in a dormant dimer, which contains, again, protected/masked functionalities identical to the original dormant monomer (Figure 1). Repeating this simple protocol results in the creation of architecturally defined structures with an exponential gain in polymer length. The IEG method has been applied extensively to synthesize flexible linear polymers (Barnes et al., 2015; Huang et al., 2017)—for example, *via* copper-catalyzed azide–alkyne click (CuAAC) reactions. However, it still remains a challenge to efficiently utilize arylation chemistry with an IEG-reaction framework, as is required in order to access conjugated or heteroatom-linked polyaromatic polymers with IEG methodology. In particular, while transition metal–catalyzed arylation reactions have been utilized previously to synthesize conjugated and/or heteroatom-linked polyaromatic macromolecules (Figure 1), (Zhang et al., 1992; Louie and Hartwig, 1998; Sadighi et al., 1998; Liess et al., 2006), there are (to the best of our knowledge) currently no transition metal-free reactions available to achieve exponential polymer growth for conjugated or heteroatom-linked polyaromatic structures. We are now able to meet this synthetic challenge by marrying iterative exponential polymer growth with nucleophilic aromatic substitution (S_N_Ar) reactions (Beugelmans et al., 1994; Blaziak et al., 2016; Landovsky et al., 2019) with a unique masking/unmasking technique. Unmasking was achieved by simply oxidizing aromatic sulfides to sulfones—a mild, simple, and scalable reaction well suited for IEG applications. At the same time, the IEG coupling reactions also proceeded under mild conditions (mild heating to ∼50°C), which represents an advantage over transition metal–catalyzed alternatives [which tend to require stronger heating (Sadighi et al., 1998)].

Our new synthetic technology for precision polymer growth helps resolve a number of concerns, which exist with traditional, transition metal–catalyzed cross-coupling methodology (Sun and Shi, 2014; Li et al., 2020). These issues include, but are not limited to 1) the inherent toxicity of many transition metal catalysts, which goes hand in hand with the need to accomplish/demonstrate complete removal of any residual transition metal, especially for healthcare-related applications (which can be difficult to achieve with polymers containing many Lewis basic groups like the ones presented in this article), 2) the relatively high cost of the required transition metal catalysts and complex ligands, which are often needed for high-yielding cross-coupling reactions suitable for polymer growth. Transition metal-free cross-coupling protocols like the one presented in this article have the potential to overcome these fundamental challenges associated with transition metal–catalyzed polymer growth.

## Results and Discussion

**SCHEME 1:**
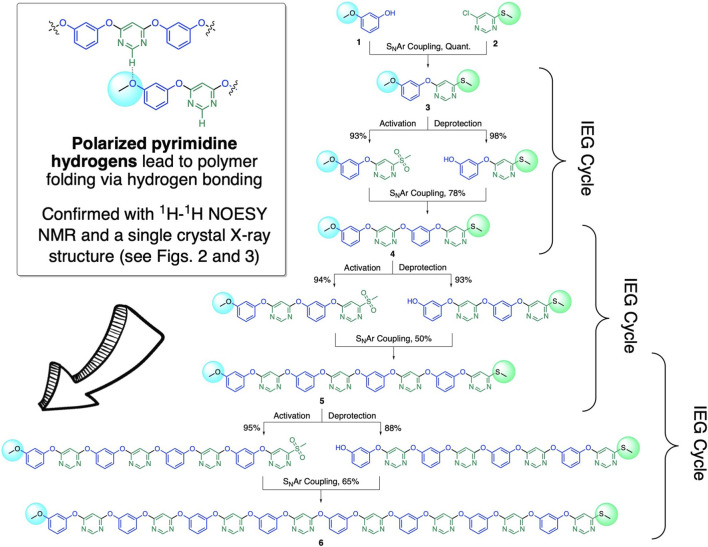
Synthesis of unimolecular polymers with alternating resorcinol and pyrimidine units with S_N_Ar-based IEG couplings, based on masking/unmasking *via* methyl sulfide to methyl sulfone oxidation. The general reaction conditions for deprotection/activation and IEG coupling are the following: **(i)** Coupling: K_2_CO_3_, DMF, 60°C, 12 h, room temperature. **(ii)** Deprotection of terminal methoxyl groups to generate nucleophilic phenols: BBr_3_, CH_2_Cl_2_, –78°C to room temperature, 12 h. **(iii)** Activation of methyl sulfides to generate electrophilic methyl sulfones: *m*CPBA, EtOAc, room temperature, 24 h.

To implement our sulfide oxidation–driven IEG coupling strategy, we first tested a variety of sulfone leaving groups for their ability to couple with phenolate anions in our IEG scheme (for related work, see: Guan et al., 2015). We discovered that simple methyl sulfones (Figure 1) are best suited for this purpose; as with sulfone substituents larger than methyl (e.g., phenyl), we tended to get lower IEG coupling yields. For our synthetic scheme, we decided to protect the phenols with methyl groups (which can readily be removed in high yield with BBr_3_). However, it is very likely that instead of just methyl groups, alternative phenol protection groups (e.g., benzyl) can also be also be utilized in the future to further enhance the functional group tolerance of our IEG coupling scheme. To start the IEG couplings, we first condensed 3-methoxyphenol (**1**) with 4-chloro-6-(methylthio)pyrimidine (**2**) in quantitative yield under standard S_N_Ar conditions with K_2_CO_3_ as the base (Van Rossom et al., 2012). The resulting dimeric product 4-(3-methoxyphenoxy)-6-(methylthio)pyrimidine (**3**) was then split in half for the next IEG deprotection/activation/coupling cycle. The first aliquot was subjected to BBr_3_ for methoxyl deprotection, while the second aliquot was activated via oxidization to the methyl sulfone with *m*CPBA. Following these deprotection/activation steps, we were able to perform an S_N_Ar-based IEG coupling, again in the presence of K_2_CO_3_ as the base. Ultimately, by iteratively implementing (Scheme 1) our new, transition metal-free IEG methodology, we were able to access macromolecules with up to 16 perfectly alternating AB units—where A and B represent resorcinol and pyrimidine rings—for the first time in a fully unimolecular fashion.

**FIGURE 2:**
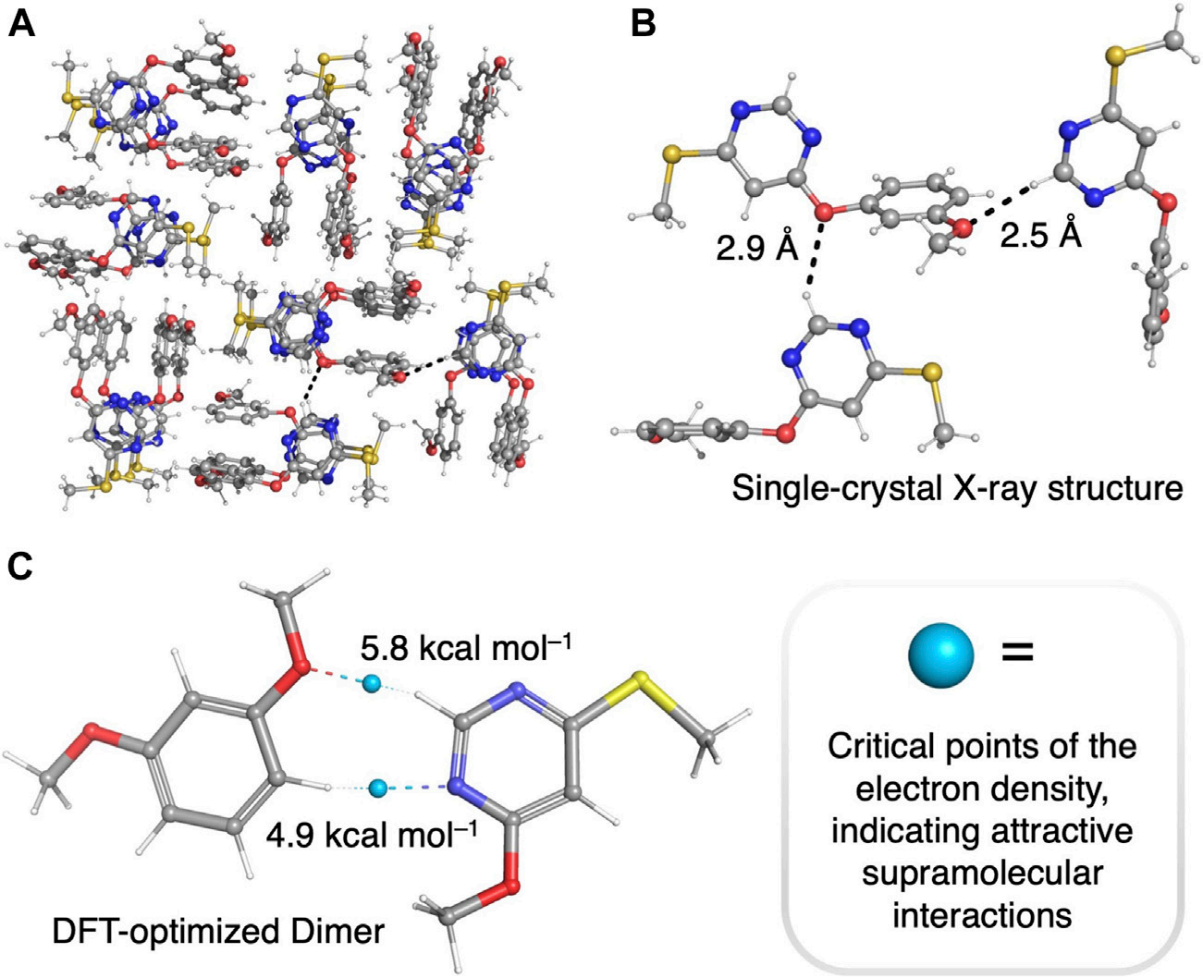
**(A, B)** Single-crystal X-ray structure of 4-(3-methoxyphenoxy)-6-(methylthio)pyrimidine **(3)**. **(C)** DFT-optimized structure (B3LYP/6-31G** level) of a model hydrogen-bonded dimer, 1,3-dimethoxyphenol in complex with 4-methoxy-6-(methylthio)pyrimidine. NCI critical points of the electron density (calculated at the B3LYP/6-31G** level of theory) are illustrated with blue spheres. Color code: C: gray; H: white; N: blue; O: red; S: yellow.

We hypothesized that the electron-deficient pyrimidine units in our unimolecular oligomers might be able to promote folding (Scheme 1, inset) *via* hydrogen bonding of and/or π-stacking with the electron-rich resorcinol units. To investigate this hypothesis, we grew single crystals suitable for single-crystal X-ray diffraction analysis of compound **3** by slow vapor diffusion of hexanes into ethyl acetate solutions of **3**. The packing observed (Figures 2A,B) upon analysis of the diffraction pattern of a single crystal of compound **3** clearly demonstrates the ability of the positively polarized -N=CH-N= hydrogens in the pyrimidine rings to form intermolecular hydrogen bonds with the methoxyl group of another molecule of **3** in the solid state. There are other hydrogen bonding interactions that are weaker, but still significant, that also contribute (Supplementary Table S1) to the supramolecular packing of this structure. To estimate the strength of the [pyrimidine–CH^…^OCH_3_] hydrogen bonds observed in the crystal structure of **3**, we optimized a model dimer—1,3-dimethoxyphenol in complex with 4-methoxy-6-(methylthio)pyrimidine—with density functional theory at the B3LYP (Lee et al., 1988; Becke, 1993)/6-31G** level of theory. Next, we used the noncovalent interactions (NCI) code (Johnson et al., 2010) implemented in the Jaguar (Bochevarov et al., 2013) software package to find all major attractive supramolecular interactions present in our model dimer, characterized as critical points of the electron density. The NCI critical points of the DFT-optimized structure of the model dimer (Figure 2C) clearly show the presence of a [pyrimidine–CH^…^OCH_3_] hydrogen bond, with a strength of ∼5.8 kcal/mol.

**FIGURE 3:**
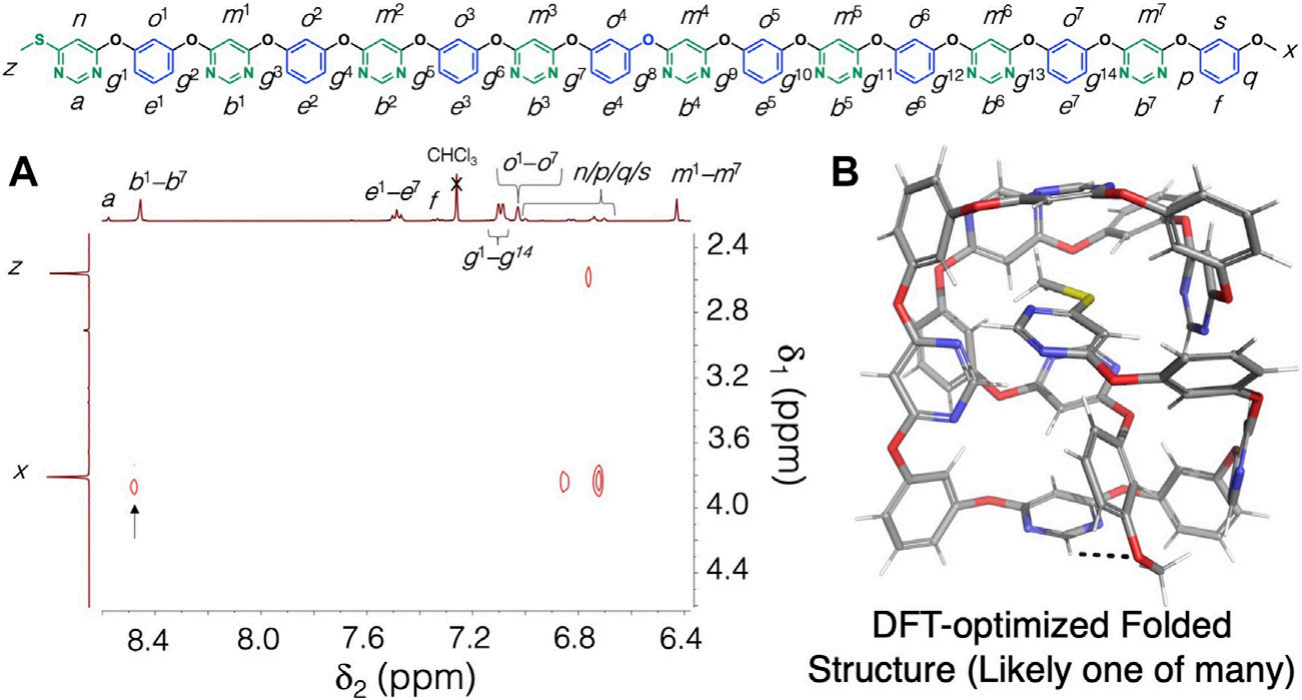
**(A)** Partial ^1^H-^1^H NOESY NMR (500 MHz, CDCl_3_, 298 K) of the hexadecamer **6**. The key NOE cross peak, which is consistent with polymer folding, is highlighted with an arrow. **(B)** The corresponding model of a potential folded structure of compound **6**. The structure shown represents the lowest energy conformation found with a MacroModel conformational search (OPLS3e force field), which was then refined with a DFT optimization at the B3LYP-D3/6-31G** level of theory.

Having established that the pyrimidine rings are attracted to the resorcinol units in the solid state via [CH^…^O] hydrogen bonding, we next embarked on investigating the ability of the polymer **6**—the longest unimolecular, alternating pyrimidine/resorcinol polymer synthesized to date—to fold, driven by [CH^…^O] hydrogen bonding interactions. To demonstrate folding, we recorded a ^1^H-^1^H NOESY NMR spectrum of the hexadecamer **6** in CDCl_3_ at 298 K. The ^1^H-^1^H NOESY NMR spectrum displays (Figure 3A) a NOE cross peak between the proton resonance *x* of the terminal methoxyl group and the -NCHN- proton resonances (*b* ^1^–*b* ^7^, which all overlap at the same resonance frequency at 8.48 ppm). Notably, this NOE cross peak is absent in the ^1^H-^1^H NOESY NMR spectrum of the dimer **3** (Supplementary Figure S1), which demonstrates that folding and/or supramolecular aggregation (and not just simply the fact that the proton corresponding to resonance *b* ^7^ is present in a pyrimidine ring next to the terminal methoxyl group) is responsible for the observed NOE cross peak. Furthermore, a ^1^H DOSY NMR spectrum of **6** (Supplementary Figure S10) confirmed that the compound does not aggregate significantly in solution, and thus the NOE cross peak observed must be stemming from intramolecular folding of the polymer chains. A folded model of the hexadecamer **6** (optimized with DFT at the B3LYP-D3/6-31G** level) is shown in Figure 3B. The structure shown represents the lowest energy conformation obtained from a MacroModel conformational search (carried out without constraints and the OPLS3e force field (Harder et al., 2016) of the hexadecamer **6**. While other potential, folded structures exist, the observed NOE cross peak is consistent with the DFT-optimized structure shown in Figure 2B, which shows a [CH^…^O] hydrogen bonding contact between a pyrimidine CH functionality and the terminal methoxyl group in **6**. For property characterization, we also performed differential scanning calorimetry (DSC) of **6** (see Supplementary Figure S11 for the DSC thermogram). As **6** is still a relatively low molecular weight compound and fully unimolecular, we did not observe any phase transitions in the temperature range investigated (30–335°C).

Overall, our results demonstrate that methyl sulfones can be effective leaving groups for transition metal-free, iterative exponential growth processes. Furthermore, the electrophilic pyrimidine cores present in our polymers not only help further enhance the yields for the S_N_Ar-based IEG coupling reactions but also provide polarized C–H bonds, capable of directing the folding of the unimolecular alternating pyrimidine–resorcinol polymers presented in this work.

## Materials and Methods

### General Methods and Materials of Synthesis

All commercially available starting materials were purchased from Sigma-Aldrich, Fisher Scientific, or Oakwood Chemical. Unless noted otherwise, all reagents were used as received without further purification. When needed, dichloromethane (CH_2_Cl_2_) and dimethylformamide (DMF) were dried using a Glass Contour solvent purification system by SG Water United States, LLC. Removal of solvents was accomplished on a Büchi R-210 rotary evaporator and further concentration was attained under a Fisher Scientific Maxima C-Plus vacuum line. Column chromatography was performed manually with Sorbent grade 60 silica with a mesh size between 230 and 400 using a forced flow of indicated solvents or automatically with a *Teledyne* CombiFlash® Rf+ chromatography system.

All ^1^H NMR spectra (see: Supplementary Figures S2, S4, S6, S8) and ^13^C (^1^H) NMR spectra (see: Supplementary Figures S3, S5, S7, S9) were recorded on a Bruker ARX 500 (125 MHz) spectrometer, and all ^1^H-^1^H NOESY NMR were recorded at 298 K on a Varian Unity Inova 500 (500 MHz) spectrometer. Samples for NMR spectroscopy were dissolved in CDCl_3_, and the spectra were referenced to the residual solvent peak (CDCl_3_: 7.26 ppm for ^1^H and 77.16 ppm for ^13^C (^1^H) NMR; or to tetramethylsilane [TMS, 0.00 ppm for ^1^H and ^13^C (^1^H) NMR]) as the internal standard. Chemical shift values were recorded in parts per million (ppm). Data are reported as follows: chemical shift, multiplicity (s = singlet, d = doublet, t = triplet, q = quartet, m = multiplet, and dd = doublet of doublets), coupling constants (Hz), and number of protons. ^1^H-^1^H NOESY NMR spectra were acquired with a NOE mixing time of 600 ms. The datasets were processed with MestReNova v10.0.2-15,465 using Bernstein polynomial fits (with a polynomial order of 3) for baseline corrections. The ^1^H DOSY NMR spectra were acquired on a Varian Unity Inova 500 (500 MHz) spectrometer, equipped with a HCN probe with *Z*-axis gradients, and a Highland Technologies L700 gradient amplifier. The standard DOSY Varian pulse program “Dbppste” was used, with a stimulated echo sequence and bipolar gradient pulse pairs. All experiments were acquired at 25°C, and DOSY spectra were processed/analyzed using Agilent’s VnmrJ (version 4.2) software, employing the discrete approach for the inverse Laplace transform in the diffusion dimension. High resolution mass spectra were obtained on a Waters XEVO G2-XS QTof in positive ESI mode. Differential scanning calorimetry (DSC) was performed on a PerkinElmer Pyris 1 differential scanning calorimeter on aluminum plates.

### General Synthetic Procedure for the IEG Deprotection/Activation and Coupling Steps

Methoxyl Deprotection: For deprotection of the terminal methoxyl groups, dormant oligomers **3, 4,** and **5** (0.224 mmol) were dissolved in anhydrous CH_2_Cl_2_ (4 ml) under an atmosphere of dry nitrogen. The reaction mixtures were then cooled to –78°C, and a 1.0 M solution of BBr_3_ in CH_2_Cl_2_ (0.896 mmol of BBr_3_) was added dropwise *via* syringe into the stirring solution. Next, the reaction mixtures were allowed to warm to room temperature over the course of ∼1 h, stirred at room temperature for an additional 12 h, cooled to 0°C, and finally quenched by adding MeOH (1 ml) and ice chips until fuming ceased. The mixtures were then added to a separatory funnel, and the organic layers were removed. The aqueous layers were extracted with CH_2_Cl_2_ (3 × 5 ml), and the combined organic layers were dried over anhydrous Na_2_SO_4_, filtered, and concentrated under reduced pressure to afford the deprotected phenols in 88–98% yield (see also Scheme 1). The free phenols were carried forward for the IEG coupling steps without further purification.

Activation of the Electrophilic Coupling Sites *via* Sulfide Oxidation: Dormant oligomers **3, 4,** and **5** (0.23 mmol) were dissolved in anhydrous EtOAc (4 ml), and a solution of 70% *meta*-chloroperoxybenzoic acid in water (0.92 mmol of *m*CPBA) was added. After stirring for 16 h at room temperature, the reaction mixtures were added to a separatory funnel, the organic layers were washed with a 0.5 N aqueous NaOH solution (5 × 4 ml) and brine (2 × 3 ml). Finally, the organic layers were dried over anhydrous Na_2_SO_4_, filtered, and concentrated under reduced pressure to afford the electrophilic methyl sulfones in 93–95% yield (see also Scheme 1). The activated sulfones were carried forward for the IEG coupling steps without further purification.

Coupling of Active IEG Units: The methyl sulfone oligomers (0.26 mmol) and K_2_CO_3_ (0.55 mmol) were added to a flame-dried, 5 ml round-bottomed flask under a nitrogen atmosphere, and anhydrous DMF (4 ml) was added. Next, the oligomers with the free phenolic ends (0.18 mmol) were added to the reaction mixtures at room temperature. Afterward, the reaction mixtures were warmed to 40°C and stirred at 40°C for 12 h. Next, the reaction mixtures were diluted with a 0.5 N aqueous HCl solution (5 ml), transferred to a separatory funnel, and extracted with EtOAc (3 × 5 ml). The combined organic phases were washed with brine (3 × 3 ml) and with a 0.5 N aqueous NaOH solution (2 × 3 ml) to ensure that any potentially remaining phenol was removed. Finally, the combined organic layers were dried over anhydrous Na_2_SO_4_, filtered, and concentrated under reduced pressure. The crude products were purified via flash column chromatography over silica gel (eluent: 0–15% ethyl acetate in hexanes) to afford the coupled IEG products **4, 5,** and **6** in a 48–98% yield (see also Scheme 1). For the hexadecameric product **6**, the yield was determined from a quantitative ^1^H NMR spectrum obtained from the crude reaction mixture (in the presence of dimethyl sulfone as the internal standard for ^1^H NMR integration) since a significant amount of material was lost during silica column chromatography purification as a result of the reduced solubility of this relatively large oligomer in the purification solvent. In general, we found that reduced solubility in hexanes of the IEG polymers with increasing lengths presented a challenge for purification of the compounds via chromatographic methods, which resulted in variable yields for the purified products.

### Single-Crystal X-Ray Crystallography

Single crystals of **3** were grown by slow vapor diffusion of hexanes into ethyl acetate solutions of **3**. Intensity data were collected on a Bruker D8 Venture kappa diffractometer equipped with a Photon II detector. An Iμs microfocus source provided the Mo Kα radiation (λ = 0.71073 A) that was monochromated with multilayer mirrors. The collection, cell refinement, and integration of intensity data were carried out with the APEX3 software (Bruker, 2018). A multi-scan absorption correction was performed with SADABS (Krause et al., 2015). The initial structure solution was solved with the intrinsic phasing methods SHELXT (Sheldrick, 2015b) and refined with the full-matrix least-squares SHELXL (Sheldrick, 2015a) program. The 1 3 5 and -2 5 4 reflections were omitted from the final refinement due to being partially obscured by the beam stop in some orientations.

Crystal data for 3: C_12_H_12_N_2_O_2_S, *M*_r_ = 248.30, crystal size 0.374 × 0.363 × 0.254 mm^3^, monoclinic, space group *P*2_1_/n, *a* = 7.9829(5), *b* = 14.2529(8), *c* = 20.6830(11) Å, α = 90°, β = 91.442(2)°, γ = 90°, *V* = 2352.6(2) Å^3^, *Z* = 8, ρ_calcd_ = 1.402 mg m^−3^, *T* = 100 (2) K, *R*_1_[*F* ^2^ > 2σ(*F* ^2^)] = 0.0288, and *wR*_2_ = 0.0790.

### Density Functional Theory

All structures were optimized with the Jaguar software package at the B3LYP-D3/6-31G** and B3LYP-D3/6-31G** levels (for the NCI critical point calculations) of theory. NCI critical points of the electron density were calculated with the Jaguar software package at the B3LYP-D3/6-31G** level.

## Conclusion

This work demonstrates a unique IEG technique, which employs nucleophilic aromatic substitution (S_N_Ar) reactions as a tool to fabricate architecturally defined oxygen-linked ABAB polymers with a metal-free coupling strategy. The monomers are designed to exhibit both nucleophilic and electrophilic character, either of which can be accessed selectively *via* deprotection of methoxyl groups and *via* oxidation of aromatic sulfides. Integrating S_N_Ar reactions with iterative exponential growth eliminates the need for expensive transition metal catalysts, which have been required so far in order to carry out iterative exponential growth of conjugated and/or unimolecular, heteroatom-linked aromatic polymers. Our transition metal-free IEG methodology allowed us to access heteroatom-linked, unimolecular aromatic polymers with up to 16 alternating resorcinol and pyrimidine units, enabling us to investigate, for the first time, the [CH^…^O] hydrogen bond–driven folding of such polymers with single-crystal X-ray crystallography, ^1^H-^1^H NOESY NMR spectroscopy, and density functional theory (DFT). Overall, our new IEG methodology advances fundamental capacity to access new unimolecular polymers needed to investigate the diverse physical and electronic properties of heteroatom-linked polyaromatic systems. We are currently applying our new transition metal-free IEG methodology to longer precision polymers with extended solubilizing chains.

## Data Availability

The crystal data for the single crystal X-ray structure reported in this paper have been deposited in the Cambridge Crystallographic Data Centre (CCDC: 2036161).
