# Supplementary material for: Iterative Exponential Growth of Oxygen-Linked Aromatic Polymers Driven by Nucleophilic Aromatic Substitution Reactions
Source: Front Chem. 2021 Apr 28;9:620017. doi: 10.3389/fchem.2021.620017 (PMC8113702; doi:10.3389/fchem.2021.620017)
Supplement: Supplementary file 1 [file DataSheet1.pdf]

Supplemental Experimental Procedures

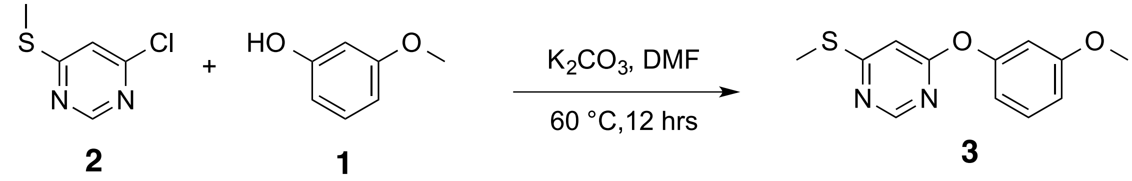

Synthesis of 4-(3-methoxyphenoxy)-6-(methylthio)pyrimidine (3): 4-chloro-6-methylthiopyrimidine (**2**, 4.00 g, 25.0 mmol) and K_2CO_3 (5.5 g, 40 mmol) were suspended/dissolved in 250 mL of anhydrous DMF in a flame-dried round-bottomed flask under a nitrogen atmosphere. Next, 3-methoxyphenol (**1**, 6.2 g, 50 mmol) was added and the solution was stirred at 60 °C for 16 hrs. Once complete, the reaction mixture was diluted with a 0.5 N aqueous HCl solution (100 mL), extracted with ethyl acetate (5 x 100 mL), and the combined organic layers were washed with a 0.5 N aqueous NaOH solution (3 x 100 mL) to ensure that all remaining 3-methoxyphenol was removed. Finally, the combined organic layers were dried over anhydrous Na_2SO_4, filtered, and concentrated under reduced pressure. The crude product was purified with flash column chromatography over silica gel (eluent: 10% ethyl acetate in hexanes) to afford 4-(3-methoxyphenoxy)-6-(methylthio)pyrimidine (**3**, 2.39 g, 9.62 mmol) as a fine powder in 97% yield.

Characterization Data for 4-(3-methoxyphenoxy)-6-(methylthio)pyrimidine (3): ^{1}H NMR (500 MHz, $CDCl_3$) δ 8.57 (s, 1H), 7.31 (t, *J* = 8.2 Hz, 1H), 6.80 (ddd, *J* = 8.4, 2.4, 0.9 Hz, 1H), 6.73 – 6.70 (m, 2H), 6.68 (t, *J* = 2.3 Hz, 1H), 6.66 (s, 1H), 3.79 (s, 4H), 2.54 (s, 4H); ^{13}C (^{1}H) NMR (125 MHz, $CDCl_3$) δ = 172.46, 168.54, 160.87, 157.89, 153.36, 130.26, 113.61, 111.57, 107.57, 103.27, 55.43, 12.84. HRMS (ESI) calcd. for $C_{12}H_{13}N_2O_2S$: *m/z* = 249.0698 $[M + H]^+$; found: 249.0692.

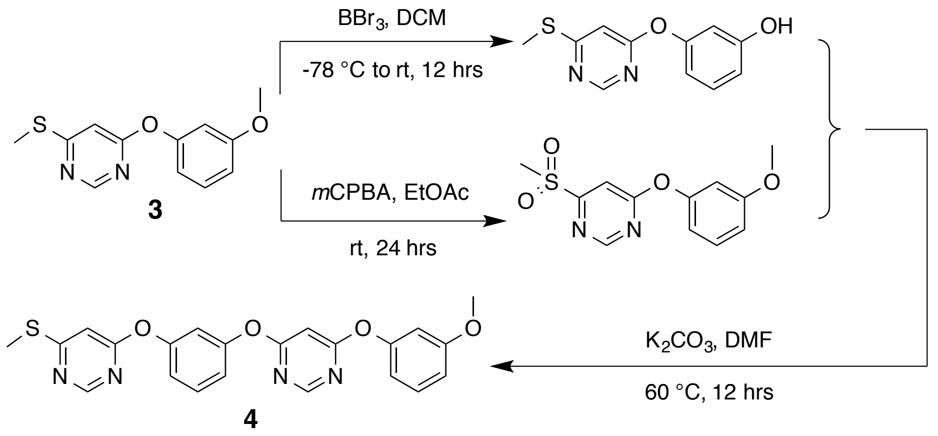

Synthesis of the Tetramer 4: The tetramer **4** was obtained in 78% yield, following the general procedure reported in Section 3.2 of the main text for IEG coupling.

Characterization data for the Tetramer 4: ^{1}H NMR (500 MHz, $CDCl_3$) δ 8.58 (d, *J* = 1.0 Hz, 1H), 8.47 (d, *J* = 0.8 Hz, 1H), 7.48 (t, *J* = 8.2 Hz, 1H), 7.34 (t, *J* = 8.2 Hz, 1H), 7.08 (dddd, *J* = 8.3, 7.4, 2.2, 0.9 Hz, 2H), 7.00 (t, *J* = 2.2 Hz, 1H), 6.83 (ddd, *J* = 8.3, 2.5, 0.9 Hz, 1H), 6.79 – 6.72 (m, 2H), 6.71 (t, *J* = 2.3 Hz, 1H), 6.36 (d, *J* = 0.9 Hz, 1H), 3.82 (s, 3H), 2.56 (s, 3H); ^{13}C (^{1}H) NMR (125 MHz, $CDCl_3$) δ = 173.09, 172.68, 171.64, 171.09, 168.10, 160.88, 158.38, 157.76, 153.40, 153.24, 153.17, 130.46, 130.27, 118.85, 118.78, 115.43, 113.61, 111.72, 107.57, 103.49, 92.47, 60.35, 55.32, 31.57, 22.64, 21.01, 14.19, 14.11, 12.82; HRMS (ESI) calcd. for $C_{22}H_{19}N_4O_4S$: *m/z* = 435.1127 [M + H]$^+$; found: 435.1123.

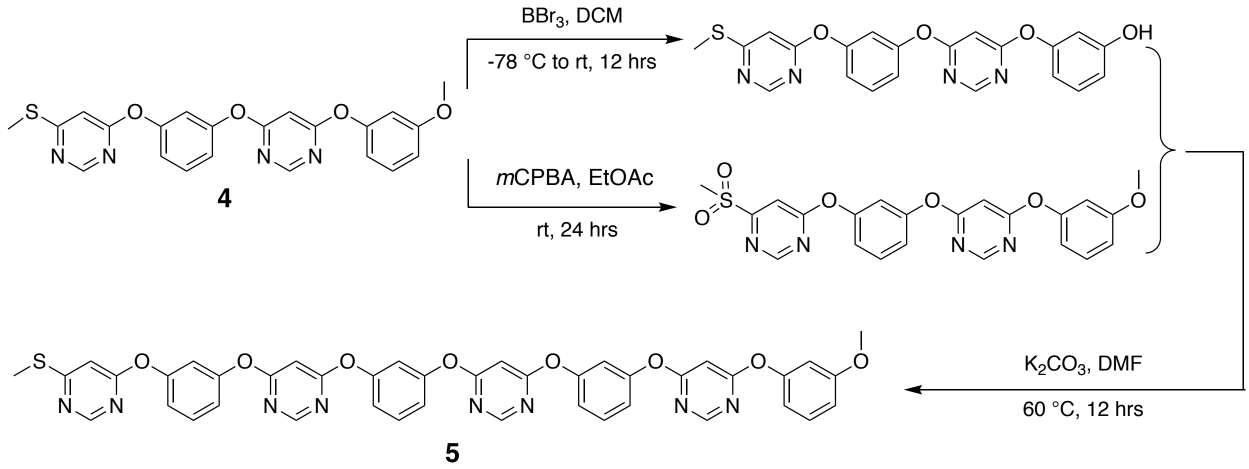

Synthesis of the Octamer 5: The octamer **5** was obtained in 48% yield, following the general procedure reported in Section 3.2 of the main text for IEG coupling.

Characterization data for the Octamer 5: ^{1}H NMR (500 MHz, $CDCl_3$) δ = 8.58 (s, 1H), 8.46 (s, *J* = 9.9, 3H), 7.48 (m, 3H), 7.33 (t, *J* = 8.2, 1H), 7.13 – 6.98 (m, 9H), 6.83 (dd, *J* = 8.3, 2.1, 1H), 6.77 – 6.72 (m, 3H), 6.43 (s, 2H), 6.36 (s, *J* = 9.9, 1H), 3.81 (s, 3H), 2.56 (s, 3H); ^{13}C (^{1}H) NMR (125 MHz, $CDCl_3$) δ = 172.71, 171.67, 171.19, 171.17, 171.08, 168.11, 160.90, 158.42, 158.32, 157.80, 153.40, 153.28, 153.22, 153.20, 153.19, 130.52, 130.49, 130.31, 118.93, 118.84, 118.77, 115.43, 115.42, 113.58, 111.66, 107.59, 103.53, 92.77, 92.75, 92.48, 55.46, 12.86; HRMS (ESI) calcd. for $C_{42}H_{31}N_8O_8S$: *m/z* = 807.1986 [M + H]$^+$; found: 807.1979.

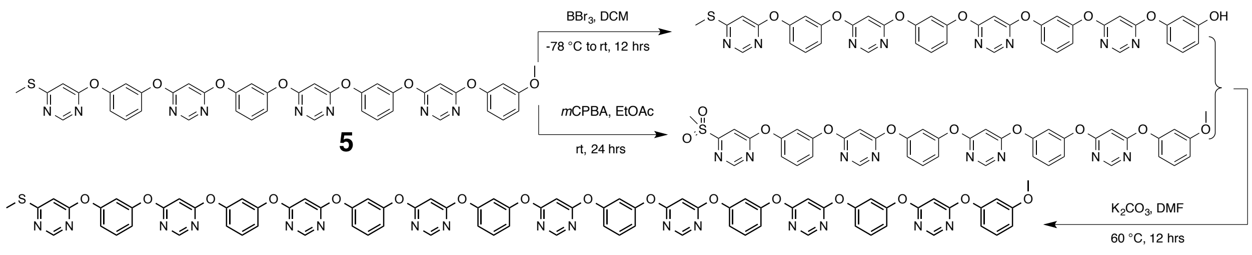

6

Synthesis of the Hexadecamer 6: Following the general procedure reported in Section 3.2 of the main text for IEG coupling, the hexadecamer **6** was obtained in 65% yield — calculated based on ^{1}H NMR integration of the *a* proton resonance present in the crude reaction product (referenced to dimethyl sulfone as the internal reaction standard).

Characterization Data for the Hexadecamer 6: ^{1}H NMR (500 MHz, $CDCl_3$) δ = 8.60 (s, 1H), 8.48 (s, 7H), 7.51 (t, J = 8.2, 7H), 7.36 (t, J = 8.2, 1H), 7.14 – 7.01 (m, 21H), 6.85 (m, 1H), 6.75–6.70 (m, 3H), 6.45 (s, 6H), 6.38 (s, 1H), 3.83 (s, 3H), 2.58 (s, 3H); ^{13}C (^{1}H) NMR (125 MHz, $CDCl_3$) δ = 172.71, 171.67, 171.08, 171.04, 168.10, 160.90, 158.43, 158.32, 157.80, 153.22, 130.52, 130.30, 118.92, 115.52, 113.58, 111.75, 111.66, 107.59, 103.53, 92.77, 92.48, 55.46, 40.71, 37.60, 29.70, 12.86. HRMS (ESI) calcd. for $C_{82}H_{56}N_{16}O_{16}S$: *m/z* = 776.1890 $[M + 2H]^{2+}$; found: 776.1913.

Supplemental Figures and Tables

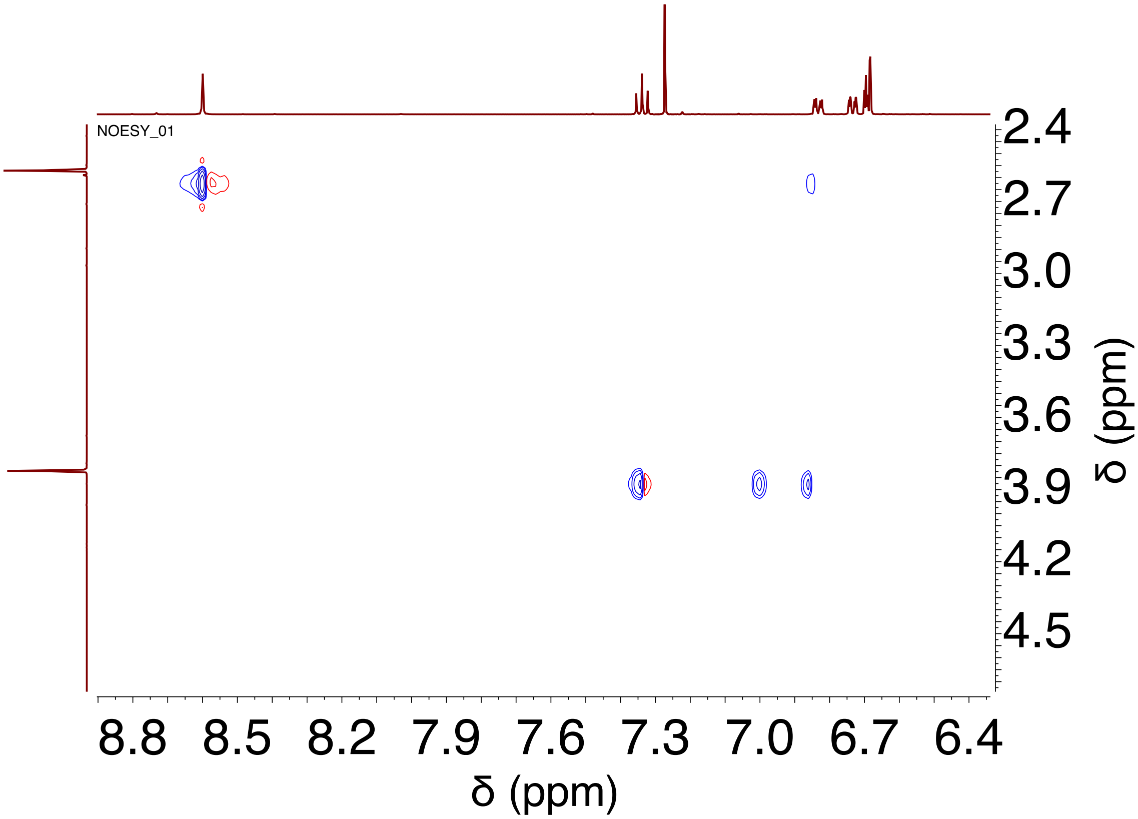

Figure S1. Partial ^{1}H-^{1}H NOESY NMR (500 MHz, $CDCl_3$) of 4-(3-methoxyphenoxy)-6-(methylthio)pyrimidine (**3**). Unlike in the case of the hexadecamer **6** (Figure 3A), no NOE cross peaks between the OCH_3 proton resonances (at 3.9 ppm) and the pyrimidine NCHN proton resonances (at 8.6 ppm) were observed.

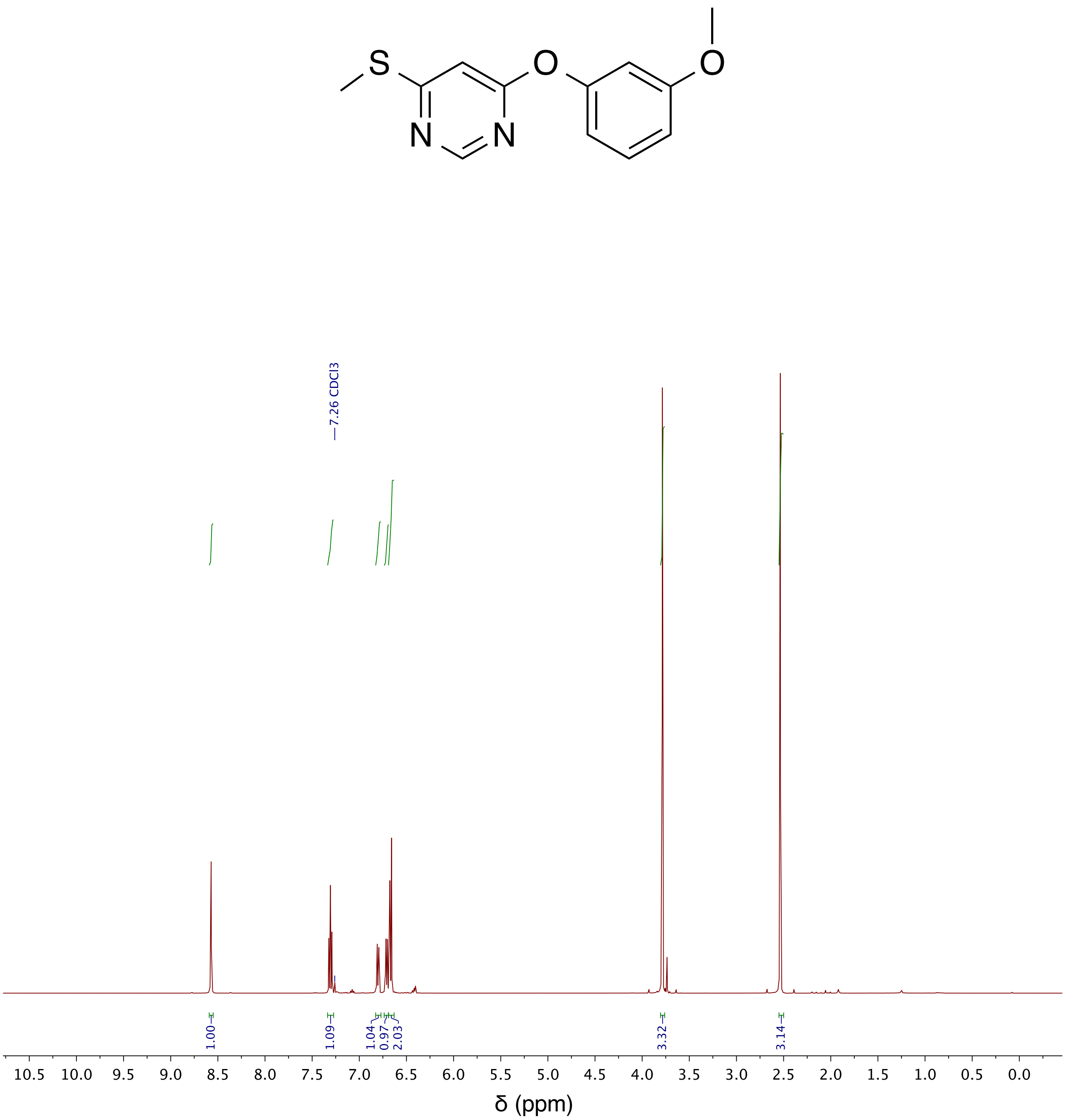

Figure S2. ^{1}H NMR spectrum (500 MHz, $CDCl_3$ 298 K) of 4-(3-methoxyphenoxy)-6-(methylthio)pyrimidine (**3**).

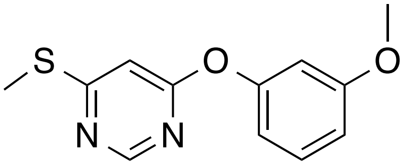

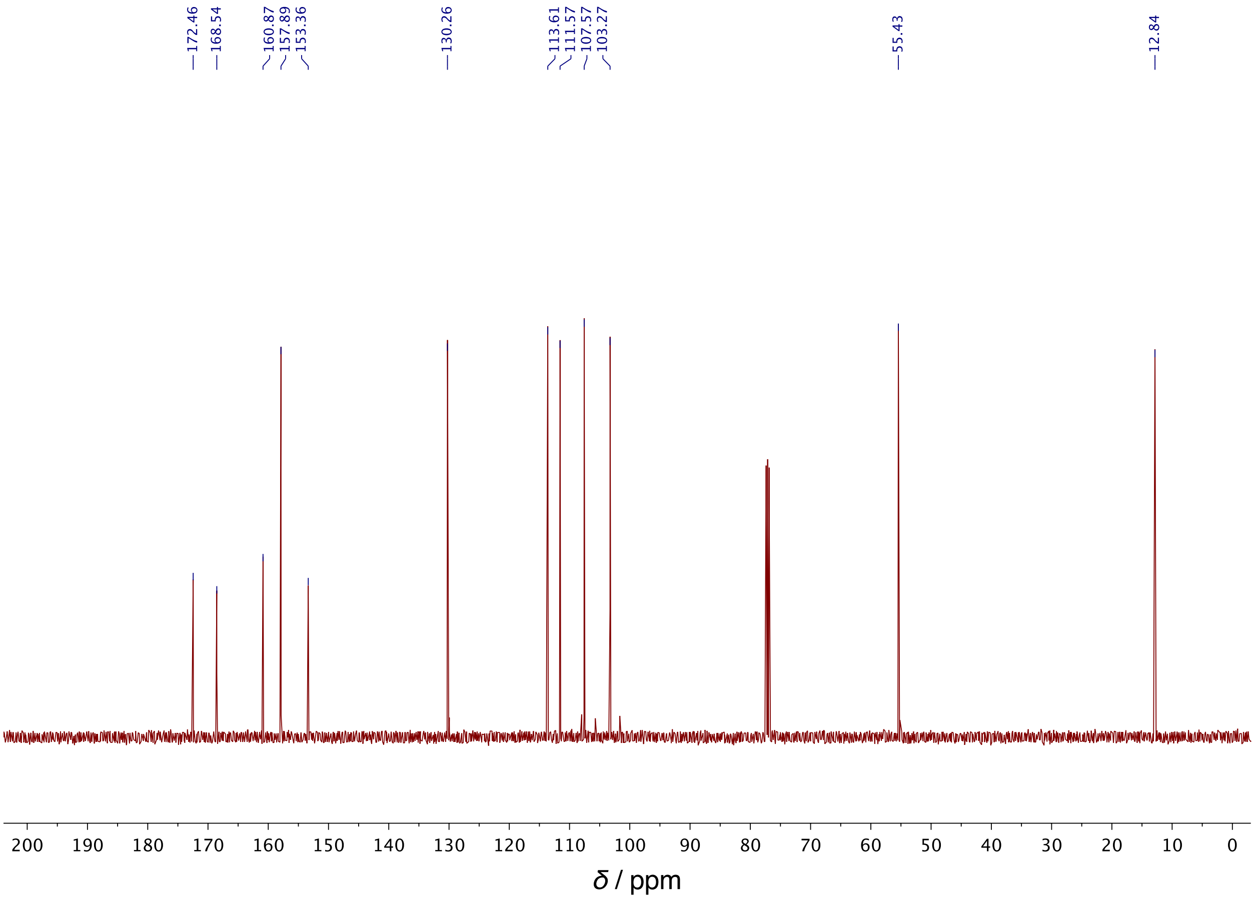

Figure S3. ^{13}C (^{1}H) NMR spectrum (125 MHz, $CDCl_3$, 298 K) of 4-(3-methoxyphenoxy)-6-(methylthio)pyrimidine (**3**).

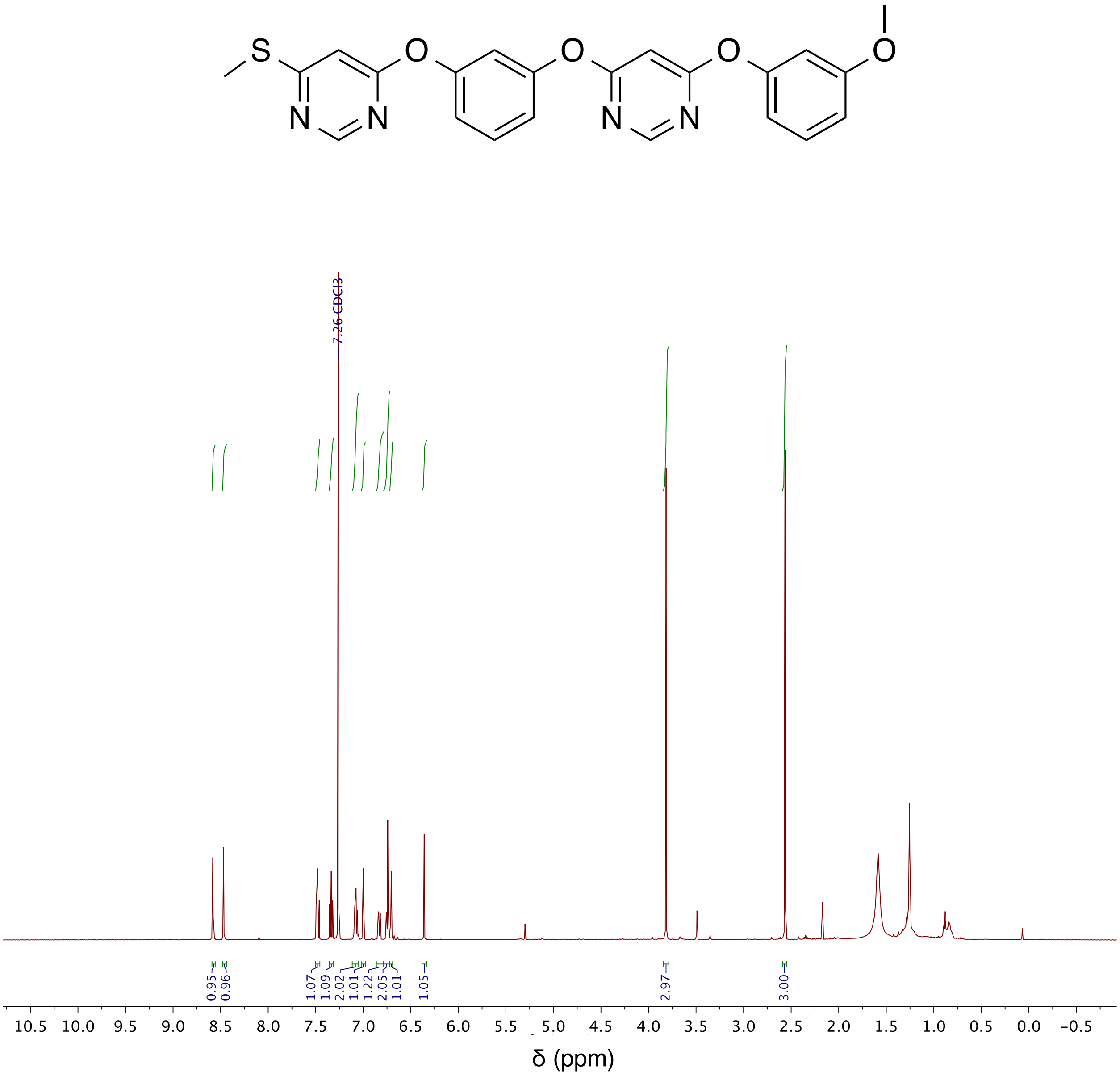

Figure S4. ^{1}H NMR spectrum (500 MHz, $CDCl_3$, 298 K) of the tetramer **4**.

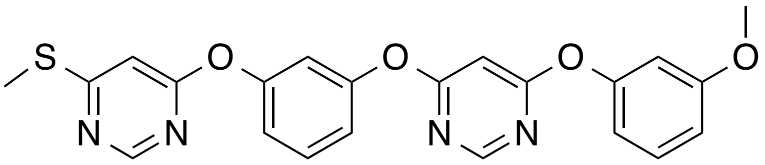

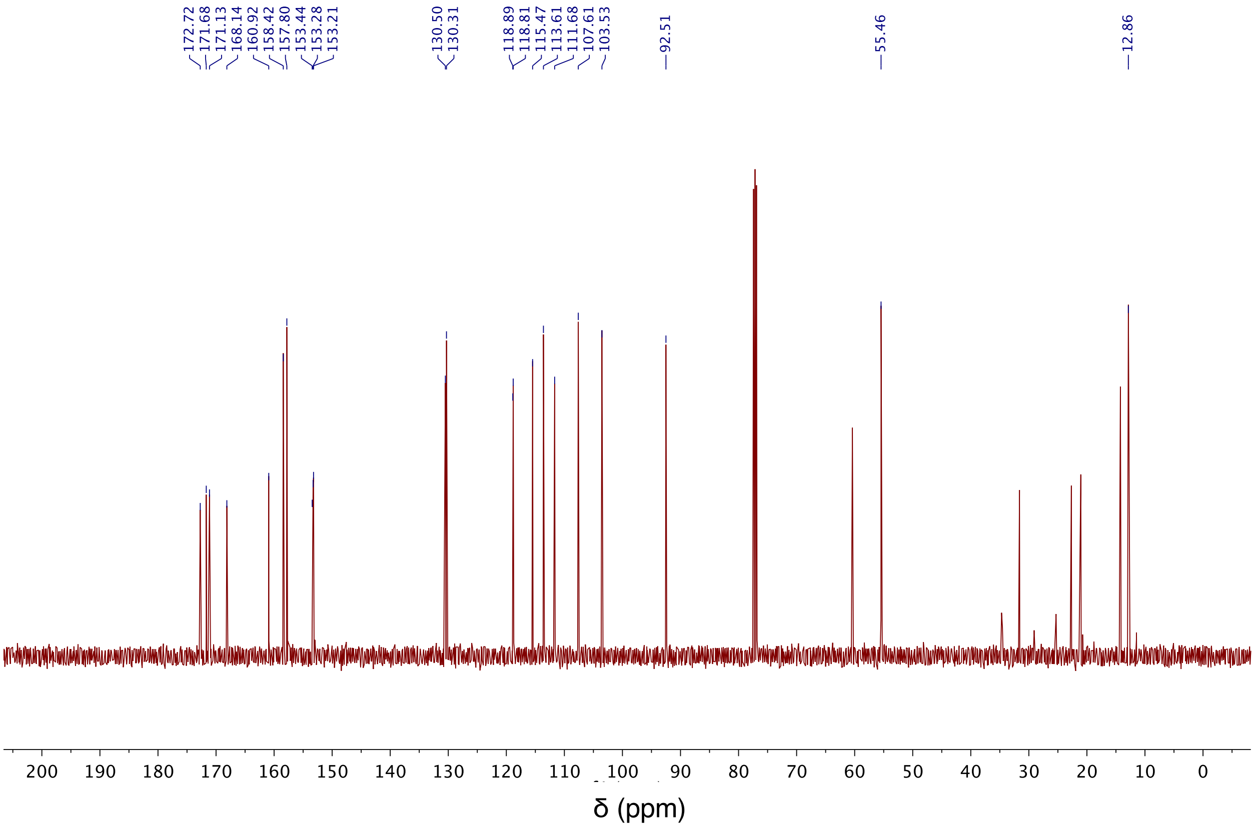

Figure S5. ^{13}C (^{1}H) NMR spectrum (125 MHz, $CDCl_3$, 298 K) of the tetramer **4**.

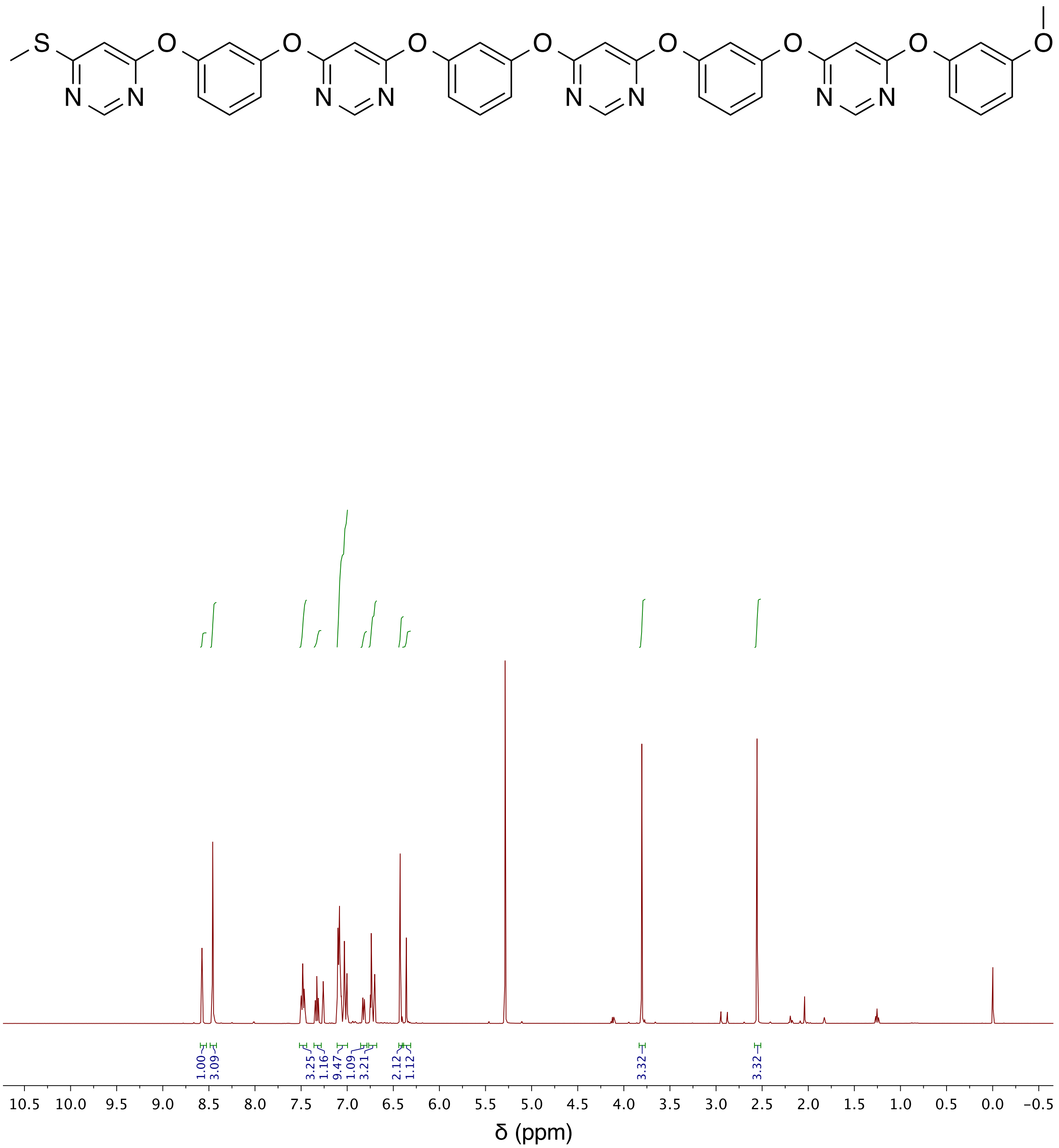

Figure S6. ^{1}H NMR spectrum (500 MHz, $CDCl_3$, 298 K) of the octamer **5**.

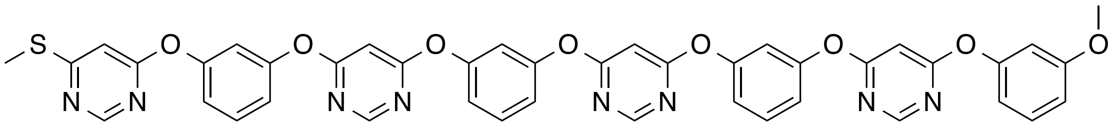

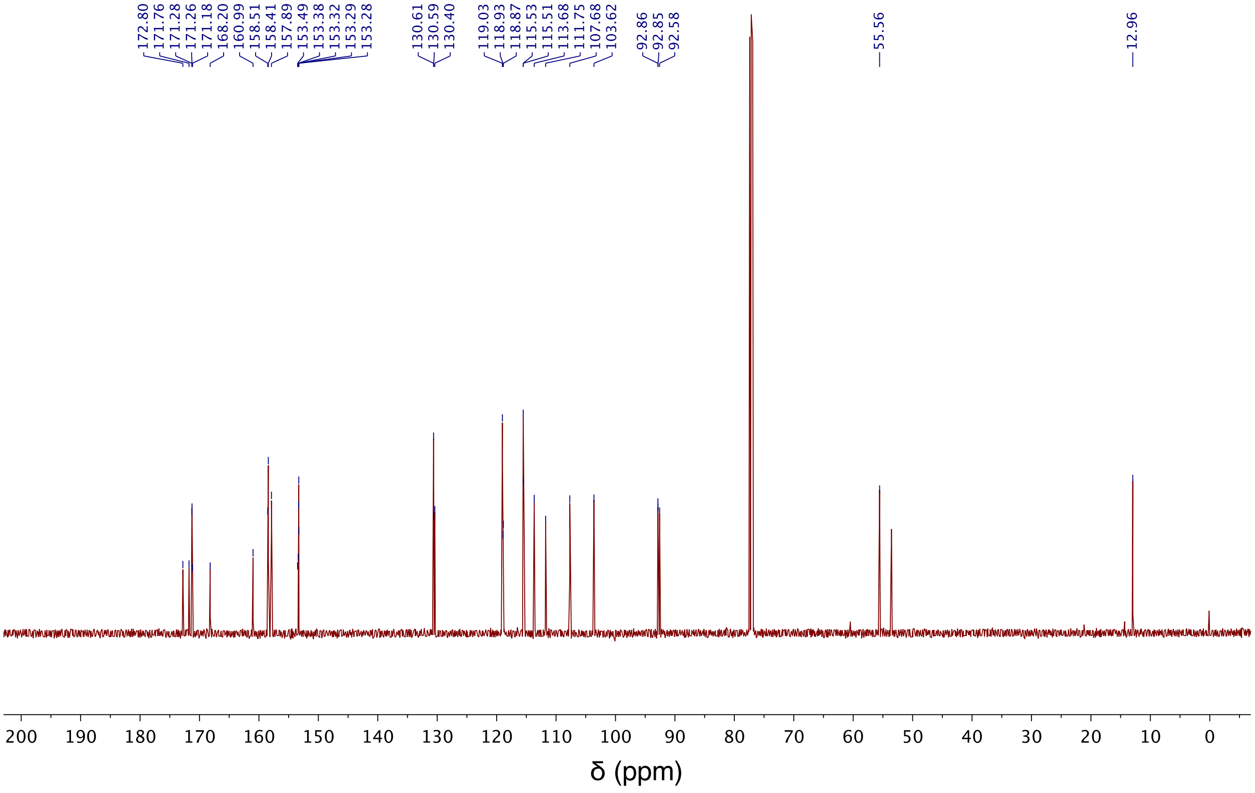

Figure S7. ^{13}C (^{1}H) NMR spectrum (125 MHz, $CDCl_3$, 298 K) of the octamer **5**.

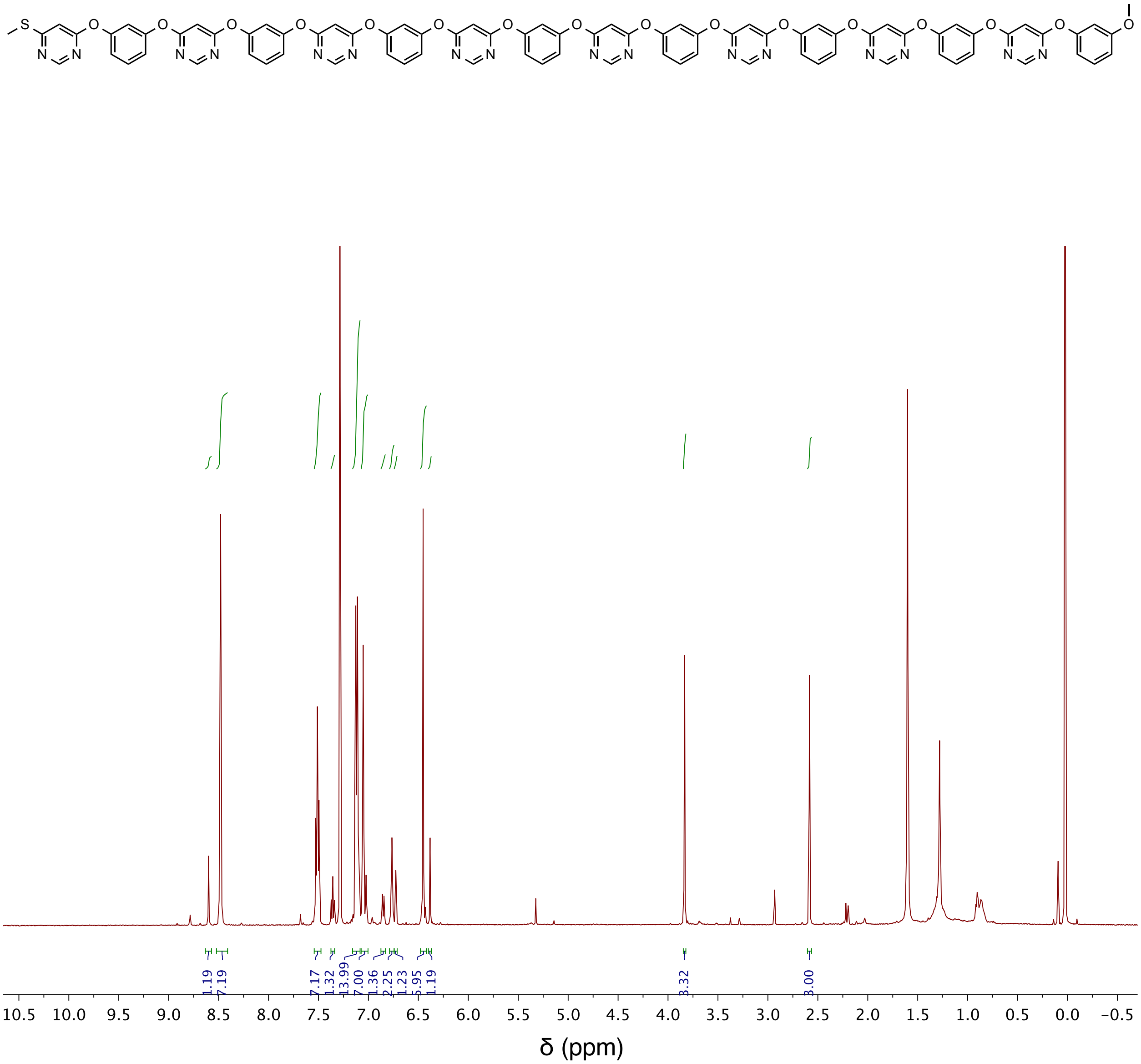

Figure S8. ^{1}H NMR spectrum (500 MHz, $CDCl_3$, 298 K) of the hexadecamer **6**.

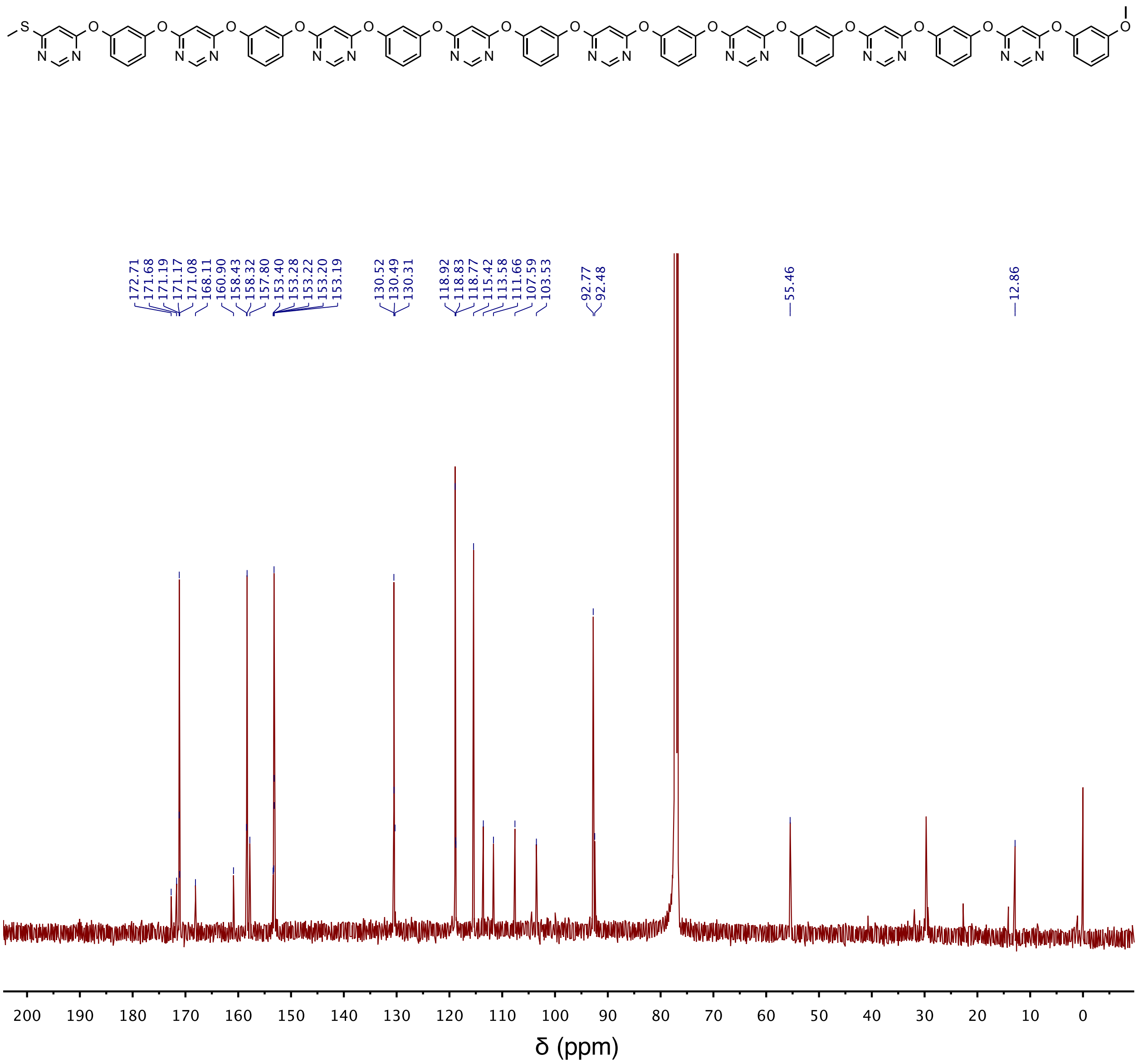

Figure S9. ^{13}C (^{1}H) NMR spectrum (125 MHz, $CDCl_3$, 298 K) of the hexadecamer **6**.

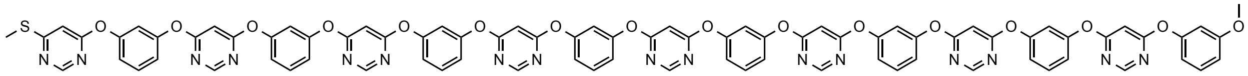

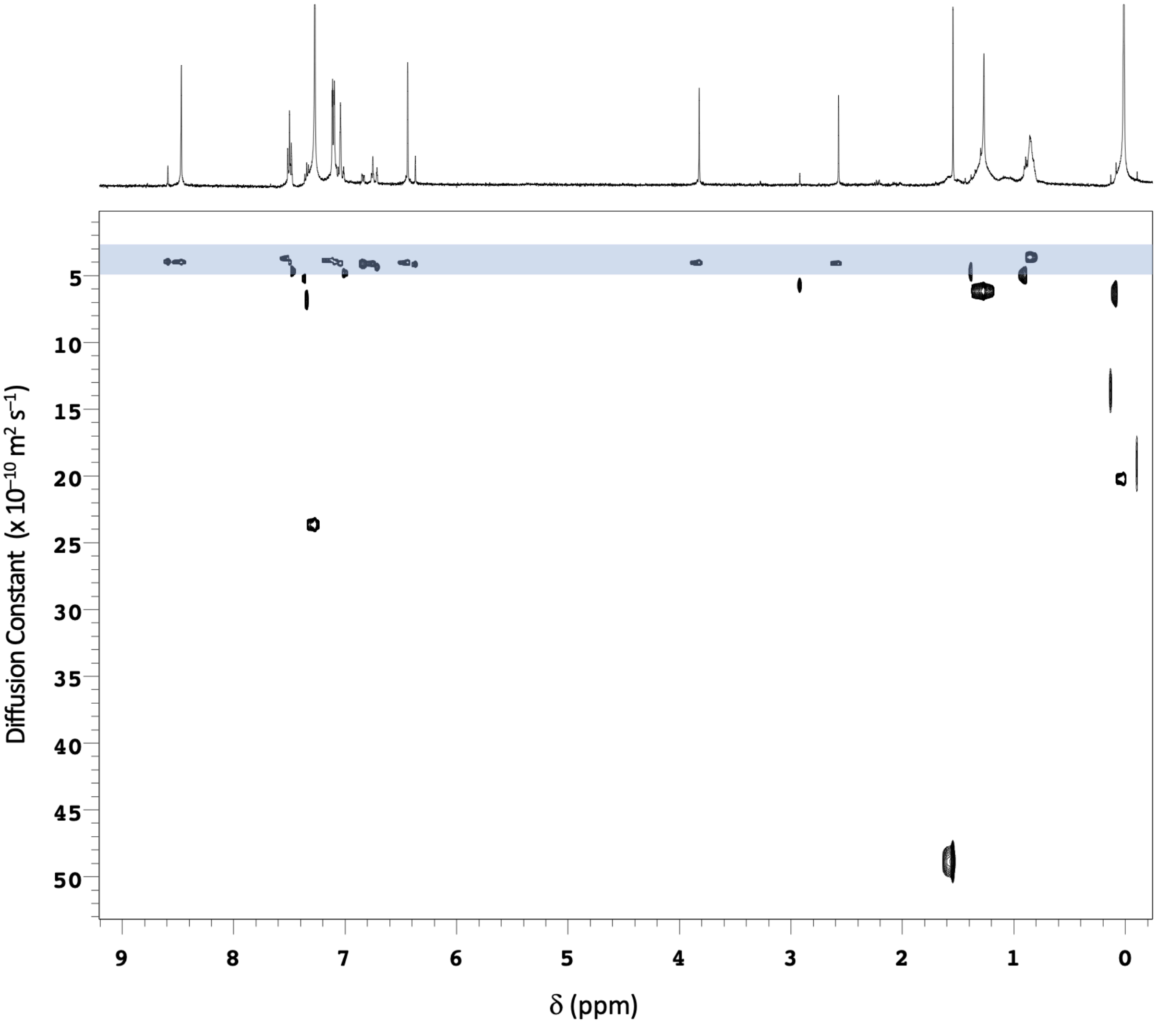

Figure S10. ^{1}H DOSY NMR spectrum (500 MHz, $CDCl_3$, 298 K) of the hexadecamer **6**.

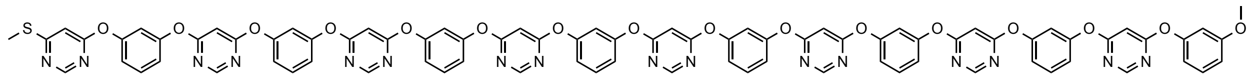

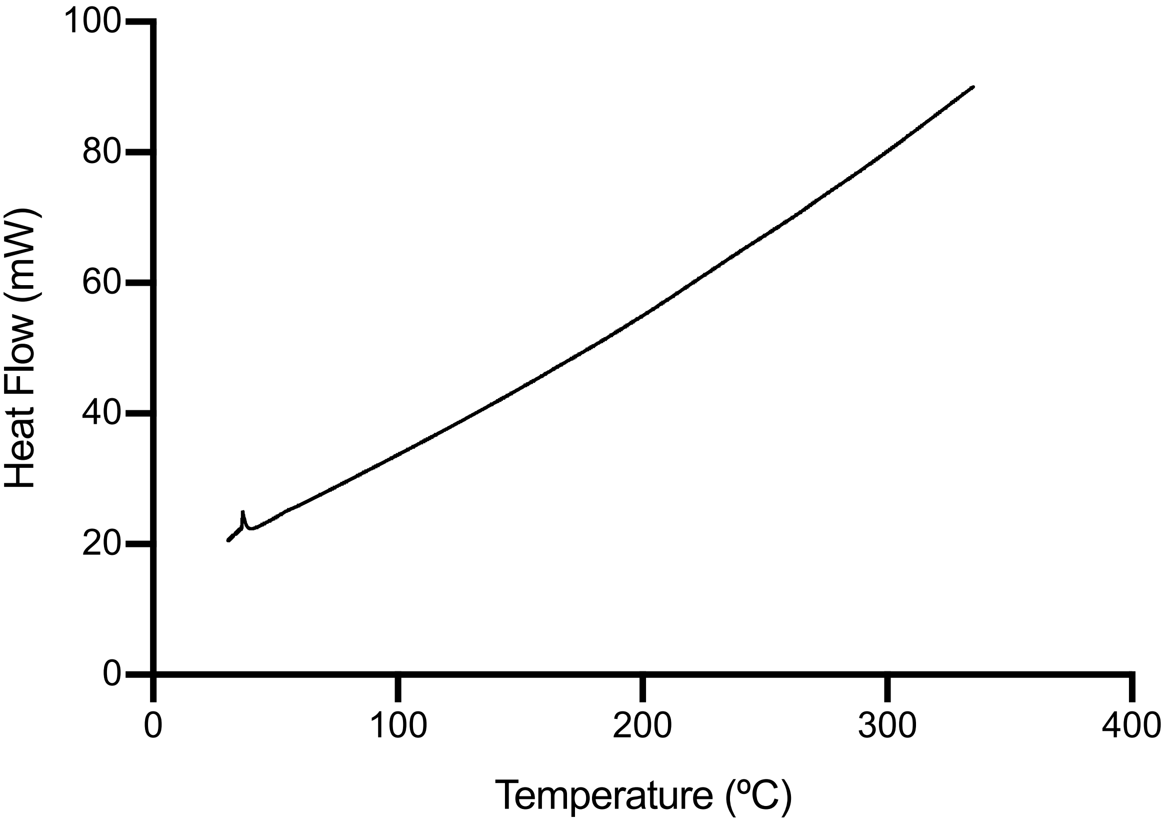

Figure S11. DSC Thermogram (1.5 mg on aluminum plate) of the hexadecamer **6**.

Table S1. Hydrogen bonds for the single crystal X-ray structure of 4-(3-methoxyphenoxy)-6-(methylthio)pyrimidine (**3**) [Å and °].

D-H···A	d(D-H)	d(H···A)	d(D···A)	<(DHA)
C(3)-H(3)...O(2)#1	0.95	2.46	3.4049(11)	171.2
C(13)-H(13B)...N(1)#1	0.98	2.65	3.5347(13)	150.7
C(13)-H(13C)...O(4)#2	0.98	2.55	3.3949(12)	144.9
C(21)-H(21)...N(1)	0.95	2.52	3.2886(11)	138.2
C(24)-H(24C)...O(2)#3	0.98	2.60	3.5063(12)	153.3

Symmetry transformations used to generate equivalent atoms:

#1 -x+1/2,y+1/2,-z+3/2   #2 -x+3/2,y+1/2,-z+3/2

#3 x+1,y+1,z
